# A multidimensional understanding of prosperity and well-being at country level: Data-driven explorations

**DOI:** 10.1371/journal.pone.0223221

**Published:** 2019-10-09

**Authors:** Mohsen Joshanloo, Veljko Jovanović, Tim Taylor

**Affiliations:** 1 Department of Psychology, Keimyung University, Daegu, South Korea; 2 Department of Psychology, Faculty of Philosophy, University of Novi Sad, Novi Sad, Serbia; 3 Interdisciplinary Ethics Applied Centre, University of Leeds, Holmfirth, England, United Kingdom; University of Bristol, UNITED KINGDOM

## Abstract

Social scientists have been interested in measuring the prosperity, well-being, and quality of life of nations, which has resulted in a multiplicity of country-level indicators. However, little is known about the factor structure of these indicators. We explored the structure of quality of life, using country-level data on tens of subjective and objective indicators. Applying factor analysis, we identified three distinct factors that exhibited both overlap and complementarity. This structure was replicated in data from previous years and with a partially different set of variables. The first factor, ‘socio-economic progress’, is dominated by socio-political and economic indicators but also includes life satisfaction, which thus appears to reflect objective living conditions. The second factor, ‘psycho-social functioning’, consists of subjective indicators, such as eudaimonic well-being and positive affective states. The third, ‘negative affectivity’, comprises negatively-valenced affective states. The three macro-factors of societal quality of life demonstrated moderate intercorrelations and differential associations with cultural and ecological variables, providing support for their discriminant validity. Finally, country and regional rankings based on the three societal factors revealed a complex picture that cautions against over-reliance on any single indicator such as life satisfaction. The results underline the need for a broadly-based approach to the measurement of societal quality of life, and provide an empirically-derived multidimensional framework for conceptualizing and measuring quality of life and well-being at country level. This study is thus an initial empirical step towards systematizing the multiple approaches to societal quality of life.

## Introduction

Well-being is an elusive construct, which defies easy and unambiguous definition. Understanding the nature and structure of well-being has been one of the challenging tasks in social science research and philosophy. At the philosophical level, there are several rival theories which make competing claims about what well-being consists in [1]. In empirical research too, a number of operationalizations, conceptualizations, and definitions of the well-being construct have been proposed across disciplines [2, 3]. We are well past the era when traditional income-based economic indicators were considered sufficient indicators of societal well-being. Humanistic and social measures (e.g., health, education, and social capital) are increasingly emphasized and incorporated in empirical research on well-being [4]. This multiplicity of theory may lead to a major challenge by hampering consensus in conceptualizing well-being both at the individual and societal levels. Yet, this multiplicity also provides a diverse dashboard of operationalizations and approaches to enable a comprehensive understanding of the complex construct of well-being while avoiding reductive and simplistic approaches [5].

We take an empirically oriented and pluralistic approach to provide an integrative and multidimensional framework for conceptualizing and appraising quality of life, prosperity, and well-being at societal level. In doing so, we bring together a relatively comprehensive array of indicators of societal quality of life which has never before been collectively investigated in a single study. Our wide-ranging suite of indicators covers subjective and objective, as well as hedonic and eudaimonic aspects of quality of life. Our results suggest that the variables group into overlapping and supplementary dimensions. We believe this empirically-based categorization of the indicators has the potential to add new depth and insight to our understanding of prosperity and well-being at the societal level.

### Measurement of societal well-being

Gaining insight into the nature of societal well-being (or quality of life, prosperity, and societal progress and development) has been inextricably linked with the question of how to measure this construct. The construct has traditionally been measured using economic indicators such as national income per capita and gross domestic product (GDP) per capita [6]. However, in recent years the inadequacy of economic indicators as proxies of well-being has been widely recognized and discussed (for an overview, see [7]). A number of non-economic dimensions of well-being had been systematically proposed, initially within the social indicators movement in the late 1960s and 1970s, to supplement economic measures as indicators of a country’s prosperity and progress [8].

Beyond the academic sphere, governments and international organizations too have increasingly recognized the need to go beyond economic measures. The UK, for example, instituted a programme entitled Measuring National Well-being, using a wide range of well-being measures, following the then Prime Minister, David Cameron’s 2010 declaration of intent to measure national progress ‘not merely by standard of living, but by quality of life’. Similarly, France has introduced quality of life measures in response to the findings of a Commission on the Measurement of Economic Performance and Social Progress [9]. Other developed countries, and international bodies such as the European Union (‘GDP and Beyond’) and the OECD (the Better Life Index) have introduced similar initiatives [10].

The predominant view in modern well-being research is that societal well-being is essentially a complex, multidimensional construct, including both objective and subjective indicators, and accordingly a comprehensive assessment of well-being must also capture both these aspects [11, 12]. The objective approach, originating in economics, focuses on material resources and objective indicators as proxies for well-being, whereas the subjective approach, originating from social survey research and psychology, concerns how individuals experience their lives and focuses on subjective evaluations of life and individuals’ inner feelings [13, 14]. The multidimensional approach to human well-being still needs to answer the question of which dimensions should be included in the conceptualization of well-being. This problem is especially evident regarding subjective indicators, due to a variety of well-being models developed in psychology over the past few decades. In general, well-being models in psychology take one (or both) of two relatively distinct, yet overlapping approaches to human well-being: the hedonic (or subjective) perspective and the eudaimonic perspective [15–17], which are discussed below.

### Hedonic/Subjective well-being

The hedonic perspective on well-being emphasizes the cognitive and affective evaluations of one’s life, and is usually operationalized as subjective well-being (SWB), which includes positive affect, negative affect, and overall life satisfaction [18]. Probably the most widely used and reported SWB indicator has been life satisfaction, which is increasingly being promoted as an alternative measure of well-being to inform public policy [19]. Consequently, most large-scale surveys of quality of life include a single-item (or longer) scale of life satisfaction as a key measure of well-being. Outside the realm of empirical social science, some philosophical theories define well-being in terms of life satisfaction [20]. Life satisfaction’s attractiveness to those who favor a simple metric of well-being can be explained by the fact that, even if it is not regarded as definitive of well-being in its own right, it can be seen as equivalent to a person’s own assessment of their well-being, taking into account other aspects [1]. Thus, while most individual measures can only plausibly capture part of the picture regarding well-being, life satisfaction has a reasonable claim to be an indicator of a person’s overall well-being.

Reliance on life-satisfaction as the sole measure of well-being rests on two assumptions: a) that a score on a life-satisfaction scale accurately reflects a person’s assessment of their own well-being; and b) that a person is a reliable judge of their own well-being. Both of these assumptions are open to question. There is evidence that life-satisfaction scores (and indeed, other SWB measures) adapt to circumstances, an effect first identified by Brickman, Coates, and Janoff-Bulman [21], and are susceptible to a variety of influences which are arguably unrelated to well-being, such as social comparisons [22] and ethical norms [23]. There is considerable debate about the extent to which these effects undermine subjective well-being measures, if at all [24, 25].

In measuring well-being some authors have exclusively focused on subjective indicators. For example, the Australian Unity Well-Being Index measures well-being by merely assessing satisfaction across several aspects of life [26]. SWB, particularly life satisfaction, has been advocated by some researchers as the gold-standard measure for assessing well-being [27]. However, many researchers have criticized this approach as arbitrary, simplistic, and reductive [5, 28]. Diener, Inglehart, and Tay [29] point out that “… life satisfaction measures have clear limits, and provide only one type of information to policymakers.” (pp. 521–522). In her review of societal well-being variables, Delle Fave [30] concludes that “there is no consensus on which subjective variables could be the ideal and most reliable indicators of well-being” (p. 85). In the present article, we take the view that, whilst life satisfaction remains a useful measure of well-being, it would be unwise to rely solely upon this single measure and that a broader mix of subjective and objective measures is more appropriate to measure national well-being and quality of life. In fact, there is increasing recognition of the need to integrate hedonic and eudaimonic approaches to well-being rather than relying solely on one and dismissing the other [15, 31].

### Eudaimonic well-being

Eudaimonic well-being concerns psychological and social qualities that make a life worth living [32]. Most definitions of eudaimonic well-being exclude emotional components and instead focus on the fulfillment of human potential, psycho-social skills, a meaningful life, and self-actualization [33, 34]. Ryff’s [35] model of psychological well-being, which includes six key dimensions of autonomy, personal growth, self-acceptance, purpose in life, environmental mastery, and positive relationships, seems to be currently the most dominant in the field of eudaimonic well-being at the individual level of analysis. Other eudaimonic models posit highly similar qualities and skills [36].

The majority of previous global studies on well-being and discussions on the policy implications of well-being have focused on SWB indicators, especially life satisfaction [37]. Eudaimonic well-being measures have been rarely used in global well-being studies and largely ignored in debates on public policy. This seems to be largely due to the fact that comparable multinational data on eudaimonic well-being are only recently beginning to emerge. Huppert and So [31], provided a preliminary examination of eudaimonic well-being based on the data collected in the sixth round of the European Social Survey in 29 European countries. Joshanloo [33] developed a new index of eudaimonic well-being across 166 countries (capturing learning experience, social support, respect, efficacy beliefs, sense of freedom, and pro-sociality), which makes global studies of eudaimonic well-being possible. Individual-level studies suggest that hedonic and eudaimonic aspects are related, yet distinct components of mental well-being [16]. Joshanloo’s [33] results replicate this finding at the societal level. Thus, there is a strong case for including these both hedonic and eudaimonic aspects in holistic models of well-being since they capture different components of human well-being [38] and have differential relationships with other variables [39].

### Measurement of societal well-being

Although most studies of well-being have focused on individual differences, the assessment of well-being at the country level has also flourished over the past few decades [40]. Although it is a widely researched topic in recent years, there is no consensus about the conceptualization and measurement of societal well-being. Different organizations and authors across various fields have proposed an array of dimensions and composite indicators in order to measure progress towards valuable goals and to enable informative and meaningful cross-country comparison [41].

One of the first attempts to measure material and social prosperity via multiple dimensions was made by the United Nations Development Programme (UNDP), when it introduced the Human Development Index (HDI) in 1990. The HDI is probably the best known composite well-being index, capturing three dimensions: longevity, education, and income. However, the HDI has received some criticisms (for an overview, see [42]); the main criticism being its narrow definition of well-being and the inclusion of too few dimensions. Thus, a number of alternative approaches to the measurement of prosperity and well-being at the national level have been developed in recent years, in order to capture additional relevant aspects of human well-being not included in the HDI. For example, in an effort to select and study quality of life variable in a systematic way, Diener [43] developed a value-based index of societal quality of life, where variables selected for measuring quality of life are reflective of prominent values endorsed across societies. This value-based index of quality of life is grounded on the universal structure of values constructed by Schwartz [44], with two proxy variables measuring each of the seven universal values of hierarchy, mastery, affective autonomy, intellectual autonomy, egalitarian commitment, harmony, and conservatism.

The Legatum Prosperity Index, first launched in 2007, measures human progress by assessing nine dimensions: Economic Quality, Business Environment, Governance, Personal Freedom, Social Capital, Safety and Security, Education, Health, and the Natural Environment (for a detailed description of the dimensions, see [45, 46]). The HDI and Legatum Prosperity Index are closely related and reliable measures [47], but although they share approximately 75% of their variance, it has been argued that the Legatum Prosperity Index is a more holistic and comprehensive measure than the HDI [45]. Other examples include the Better Life Index [9, 48] and the Social Progress Index (SPI; [49]), which are available for a smaller number of countries.

There are many other models and measures of national well-being (as reviewed, for example, in [50]). Despite notable differences in approaches to the measurement of societal well-being and prosperity, there are also important similarities between different approaches. For example, Phillips [28] provides an integrative review of the societal quality of life models and identifies four common components: civic integration, equal status (e.g., reciprocity, trust, and tolerance), sustainability, and social cohesion.

### The purpose of the present study and variable selection

Our interest is in how well a nation is doing in a broad range of domains that reflect various aspects of national prosperity and quality of life. An important limitation of previous studies on societal prosperity and well-being is a lack of attention to the factor structure of the indicators at the societal level, typically including a limited range of dimensions, and focusing on the individual level of analysis. By including a wide range of indicators, both subjective and objective, the present study is expected to provide a more comprehensive evaluation of the structure of prosperity at societal level than previous studies. Our primary aim was to reduce the large array of quality of life variables to a smaller number of derived variables. Thus, we factor analyzed the national indicators to identify the underlying factors or dimensions of societal quality of life.

As previously noted, there is a wide range of views about which dimensions should be included when measuring prosperity. It is clear that not all aspects can be included in a single study, so every approach to selecting the key dimensions has some limitations. Here, we draw extensively upon both personal and societal well-being research but our sole focus is upon the national, rather than the individual level. Thus, we include both objective (such as income inequality and suicide rates) and subjective (such as life satisfaction and eudaimonic well-being) indicators. Our subjective indicators are aggregations of countries’ subjective judgments (as reflected in global surveys). Many of the indicators we include (such as the prosperity indices) are calculated based on both subjective and objective data.

In addition to our commitment to include both subjective and objective measures, we used three other criteria for the selection of component variables. First, given the emphasis on the complementarity of hedonic and eudaimonic aspects, we intended to include indicators of both aspects of well-being. Second, we were committed to including not only positive well-being variables (e.g., life satisfaction, positive affect, and eudaimonic well-being) but also negative mental health variables (i.e., negative affect, depression, anxiety, and suicide). Grinde [51] argues that the mammalian brain has a positive mood as a default setting, so activation of negative feelings is likely to be the main cause of low well-being. Therefore, negative emotions and mental disorders are as important as positive indicators of well-being. Finally, we only included dimensions available in most countries of the world, to maximize the global coverage of the analysis.

Based on these criteria, an initial set of 24 indicators was developed, which are introduced in the methods section and listed in Table 1. Using the data from the Gallup World Poll (GWP), we included the SWB measures of life satisfaction (both present and future), positive affect (emotions: enjoyment, rest, smile/laughter), and negative affect (emotions: stress, sadness, worry, and anger). We also included the newly developed GWP-based measure of eudaimonic well-being [33]. To complement hedonic and eudaimonic well-being indices from the GWP, we also included: (a) eight dimensions of prosperity as measured by Legatum Institute [45, 46]: economic quality, business environment, governance, personal freedom, safety and security, education, health, and natural environment; (b) three proxy measures of mental health: the disability-adjusted life years (DALYs) for depression and anxiety, two frequent mental disorders, and suicide rate; (d) income inequality measured through the Gini Coefficient; (e) globalization measured with the KOF Globalization Index, which assesses three dimensions of globalization: economic, social, and political; (f) friendship opportunities, as an important aspect of social capital and interpersonal relationships.

**Table 1:** Results of exploratory factor analysis (2015–2017, N = 128).

	Factor 1	Factor 2	Factor 3
Globalization	.**939**	-.068	-.023
Education	.**869**	-.032	-.135
Health	.**866**	.037	-.045
Safety/security	.**845**	-.086	-.088
Economic quality	.**828**	.121	-.105
Governance	.**823**	.188	.023
Business environment	.**794**	.209	-.080
Life satisfaction (ladder)	.**770**	.252	-.021
Anxiety*	.**754**	.008	.354
Personal freedom	.**707**	.290	.120
Natural environment	.**627**	.322	.056
Depression*	.**573**	-.278	.052
Smile/laughter	-.073	.**890**	.086
Eudaimonic well-being	.139	.**797**	.156
Future life satisfaction	.028	.**724**	-.089
Well-rested	-.066	.**701**	-.237
Enjoyment	.310	.**604**	-.132
Income inequality*	-.**467**	.**526**	.152
Friendship opportunities	.372	.**483**	.113
Stress	.130	.136	.**725**
Sadness	-.342	-.089	.**692**
Worry	-.217	-.032	.**684**
Anger	-.306	-.271	.**533**
Suicide*	-.140	-.009	-.302

*Note*. Factor loadings > .4 are shown in boldface.

* Variables with unexpected loadings.

We chose the Legatum Prosperity Index over other prosperity models due to its comprehensiveness (covering nine prosperity domains), its inclusion of both subjective and objective data, and its wide geographical (149 countries) and time (since 2007) coverage. However, we excluded the social capital index, given its large overlap with the eudaimonic well-being index. Yet, we included as a separate variable a GWP item used in the social capital index to capture friendship opportunities. We also included measures of income inequality and globalization, which are two other important indicators capturing societies’ functioning [52–54]. It is important to point out that our assortment of variables cover all the important aspects of societal quality of life emphasized in Phillips’s [28] and others’ integrative reviews of the literature. For example, whereas many models of societal prosperity and well-being have ignored environmental sustainability, we measure sustainability via one of the sub-indices of the Legatum Prosperity Index. The natural environment sub-index measures the quality of the natural environment, environmental pressures, and preservation efforts [45, 46].

In addition to examining the structure of prosperity at the national level, the present study also included a number of external variables to establish the nomological networks of the emerging prosperity factors, and examine their discriminant validity. There is ample evidence to suggest that various indicators of well-being have differential associations with other variables. For example, Helliwell, Huang, Wang, and Shiplett [55] found that whereas per-capita income and healthy life expectancy had significant effects only on life satisfaction, freedom and generosity had larger influences on positive affect than on life satisfaction. Diener, Ng, Harter, and Arora [56] found that economic factors (e.g., income, luxury conveniences) were consistently better predictors of life satisfaction than of affective well-being, whereas socio-psychological factors (e.g., fulfillment of psychological needs) were better predictors or affective well-being than life satisfaction. Corruption at the national level has been also found to be more strongly associated with life satisfaction than with positive and negative affect [57]. Therefore, we expected the emerging factors to exhibit differential relationships with external variables. We expected this analysis, not only to establish the discriminant validity of the emerging dimensions, but also to provide additional insights into the nature of these dimensions.

Drawing on prior research, we included a wide range of factors that have been found to affect national well-being, including socio-economic [58], cultural [59], demographic [60] and ecological [61] factors, namely, Hofstede’s dimensions of national culture [62], Inglehart–Welzel cultural dimensions [63], religiosity [64], urbanization [65], national age [66], and indices of thermal climate [67]. In selecting the external variables, we were guided by recent major reviews of the literature [68, 69].

In sum, we aimed at exploring the factor structure of the 24 prosperity and well-being indicators at the societal level and to establish the nomological network of the emerging factors. The general predictions of the present study were: (1) that prosperity will have a multidimensional structure at the national level, and the indicators would form distinct factors, and (2) that different dimensions would show interpretable and differential associations with the external cultural, demographic, and ecological variables.

## Methods

### Participants

The Gallup World Poll (GWP) dataset between 2015 and 2017 was used to construct the subjective country-level indices. Using randomly selected and nationally representative samples, GWP continually surveys residents in a large number of countries. The final sample of the study consisted of 457,129 individuals in 153 countries for whom data were available on the variables of the study. The average age in the whole sample was 41.72 (*SD* = 17.77, min = 15, max = 99). Women constituted 53.4% of the sample. The names of the countries, national sample sizes, gender ratios, and average age scores are reported in the supplementary material (S1 Table).

### Measures

Variables used in the factor analyses included: life satisfaction (ladder), future life satisfaction, enjoyment, worry, sadness, stress, anger, laughter/smile, eudaimonic well-being, friendship opportunities, rest, economic quality, business environment, governance, personal freedom, safety and security, education, health, natural environment, globalization, depression, anxiety, suicide, and income inequality. It is noteworthy that depression, anxiety, suicide, and income inequality were initially used as indicators in factor analysis, yet based on the initial results we decided to remove them from factor analysis and instead use them as external variables. In a replication analysis, we also included life satisfaction these days, happiness, and purpose in life based on items available in older Gallup data. Variables used as external variables to establish the nomological network of the emerging factors included power distance, uncertainty avoidance, individualism, masculinity, self-expression values, secular-rational values, national age, urbanization, heat demands, and cold demands.

Gallup-based variables included life satisfaction, future life satisfaction, enjoyment, worry, sadness, stress, anger, laughter/smile, friendship opportunities, rest, life satisfaction these days, happiness, purpose in life, and religiosity. All of the GWP variables are based on data collected between 2007–2012 and 2015–2017. We use 2015–2017 data for our main analyses, and 2007–2012 data for replicating the factor analytic findings. The study also included external variables (i.e., variables not from the GWP). All of the external variables refer to the year 2015 (or 2007 for the replication analysis), unless otherwise stated below.

#### Eudaimonic well-being

This national index [33] was used to measure optimal functioning. The scores are based on the GWP data and refer to the years 2015–2017 and 2007–2012. This index is composed of seven GWP items measuring learning experience, social support, respect, efficacy beliefs, sense of freedom, and pro-sociality (i.e., helping strangers and volunteering).

#### Other Gallup-based variables

These variables were each measured with a single item. The GWP items used for measuring these variables are shown in S2 Table in the supplementary material. The scores were averaged in each country to construct a national score for that country. Three variables of life satisfaction these days, happiness, and purpose in life were not available in the period 2015–2017, and hence were only used in the analysis based on the 2007–2012 data.

#### National prosperity

Eight out of nine sub-indices of the Legatum Prosperity Index related to 2007 and 2015 [45, 46] were used to measure various aspects of national prosperity. The nine “pillars” of prosperity include economic quality, business environment, governance, personal freedom, social capital, safety and security, education, health, and natural environment. Each of the sub-indices consists of dozens of objective and subjective variables. More information can be obtained from http://www.prosperity.com. As some of the items used to build the eudaimonic well-being index have also been used in developing the social capital index, social capital was excluded in this study (four out of seven items of the eudaimonic well-being index are shared with the Legatum social capital index, which we consider a large overlap).

It is also noteworthy that the efficacy item used in the eudaimonic well-being index is also used in the business environment subindex of the prosperity index. Some of the affect items (i.e., enjoyment, rest, smile/laughter, and sadness) have been used to construct the health subindex. However, the health subindex consists of a large number of other indicators, and more importantly in the factor analyses, these affect variables and health were found to load on separate factors. Finally, the freedom item which is used in the eudaimonic well-being index is also part of the personal freedom subindex. Again, the personal freedom subindex consists of a large number of other indicators, and eudaimonic well-being and personal freedom loaded on separate factors. Therefore, we decided not to leave out these crucial variables from the analyses to enable a holistic exploration of these important variables.

#### Globalization

The KOF globalization index [70] related to years 2007 and 2015 was used as a comprehensive measure of globalization, assessing the economic, social, and political dimensions of globalization.

#### Depression and anxiety

The 2015 data from the Global Burden of Disease Study [71] was used to measure the disability-adjusted life years (DALYs) due to anxiety and depression, including years of life lost from premature death and years lived with less than full health. [One DALY “can be thought of as one lost year of healthy life and the burden of disease as a measurement of the gap between the current health of a population and an ideal situation where everyone in the population lives into old age in full health” [72, p. 1579]. The DALY, is a combinatory metric “designed to quantitatively measure the impact of various diseases and conditions on the productivity and well-being of people through a combination of mortality and morbidity estimates” [73, p. 2].]

#### Suicide

The 2015 age-standardized suicide rate was used to measure the prevalence of suicide in each country (http://www.who.int/gho/en/).

#### Income inequality

The GINI estimates for countries were obtained from the UNU-WIDER, World Income Inequality Database (WIID3.4), which is a database of information on income inequality (https://www.wider.unu.edu). All GINI estimates available for each country between the years 2005–2015 were averaged to obtain an income inequality estimate for each nation.

#### Hofstede’s dimensions of national culture

Hofstede’s four dimensions include power distance (“the degree to which the less powerful members of a society accept and expect that power is distributed unequally”), uncertainty avoidance (“the degree to which the members of a society feel uncomfortable with uncertainty and ambiguity”), individualism (“a preference for a loosely-knit social framework in which individuals are expected to take care of only themselves and their immediate families”), and masculinity (“a preference in society for achievement, heroism, assertiveness, and material rewards for success”) [74]. The scores for each country were obtained from Hofstede, Hofstede, and Minkov [75].

#### Inglehart—Welzel cultural dimensions

Scores of self-expression/survival values (the extent to which people prioritize individual choice over survival needs) and traditional/secular-rational values (the extent to which people emphasize modern values over traditional values such as religion, absolute standards, and conventional family values) were based on the data from the World Values Survey, Wave 6 (2011–2014). For countries that did not participate in Wave 6, data from Wave 5 (2005–2008) was used [76].

#### National age

In order to measure national age, percentages of the population that are aged 65 and above in each country were used. The data were obtained from https://data.worldbank.org.

#### Religiosity

We used Joshanloo and Gebauer’s [77] Gallup-based index of religiosity. This index is calculated as the percentage of people per country who stated that religiosity is an important part of their daily lives.

#### Urbanization

In order to measure the national degree of urbanization, percentages of the urban population in each country in the year 2018 were used. The data were obtained from the world urbanization prospects at https://esa.un.org/unpd/wup/.

#### Temperature

Van de Vliert’s [78] indices of thermal climate were used to measure temperature. The hot and cold measures are based on winter and summer deviations from a biologically optimal point of reference (i.e., 22°C or about 72°F). Climatic demands “are operationalized across each country’s or region’s major cities, weighted for population size, as the sum of the absolute deviations from 22°C for the average lowest and highest temperatures in the coldest month and in the hottest month” [78, p. 470].

## Results

The analyses started with the main dataset related to 2015–2017. A principal axis factoring with promax rotation (Kappa = 2) was run using the 24 variables (the correlation matrix for the 24 variables is shown in S3 Table in an excel file). Only 128 countries with complete data were included. Scree plot suggested a 3-factor solution. The factor loadings of this initial model are shown in Table 1. Four variables demonstrated unexpected loadings. Anxiety and depression had positive loadings on the first factor. Income inequality had a positive loading on the second factor. Finally, suicide did not have a substantial loading on any of the factors. We decided to omit these four variables from the factor analysis and instead use them as external variables. This decision was driven by the fact that our aim was to identify unambiguous interpretable factors. In addition, these results cast doubt on the suitability of these variables to measure societal well-being (as further discussed in the discussion section). Thus, to maintain more intuitive factors, these four variables were removed from the analysis and factor analysis was repeated with 138 countries that had complete data on the remaining 20 variables.

Again the Scree test suggested a three-factor solution. The results are shown in Table 2. The three factors collectively explained about 68.02% of the variances in the variables. The first factor is loaded by globalization, and all aspects of national prosperity as well as life satisfaction (i.e., the ladder question). Generally speaking, this factor represents globalization and modern (vs traditional) values, as well as financial and socio-political development, and hence can be labeled as “Socio-Economic Progress”.

**Table 2:** Results of exploratory factor analysis (2015–2017, N = 138).

	Factor 1	Factor 2	Factor 3
Globalization	.**957**	-.112	-.021
Education	.**868**	-.072	-.155
Governance	.**850**	.129	.030
Safety/security	.**839**	-.111	-.088
Economic quality	.**816**	.109	-.117
Business environment	.**815**	.149	-.089
Health	.**811**	.033	-.108
Personal freedom	.**762**	.188	.150
Life satisfaction (ladder)	.**726**	.271	-.055
Natural environment	.**685**	.246	.089
Smile/laughter	-.023	.**863**	.082
Eudaimonic well-being	.137	.**814**	.140
Future life satisfaction	.026	.**735**	-.075
Well-rested	-.031	.**641**	-.241
Enjoyment	.288	.**609**	-.171
Friendship opportunities	.385	.**453**	.075
Sadness	-.280	-.064	.**763**
Worry	-.159	-.056	.**716**
Stress	.145	.125	.**644**
Anger	-.327	-.187	.**499**
Eigenvalues	9.786	2.425	1.392
% of Variance explained	48.931	12.126	6.960

*Note*. Factor loadings > .4 are shown in boldface.

The second factor is dominated by psychological and subjective variables, including eudaimonic well-being and positive affective states. With variables such as smile/laughter, eudaimonic well-being, future life satisfaction, enjoyment, and friendship opportunities, this factor measures a vital and psychosocially rich lifestyle. Thus, this factor can be labeled as “Psycho-Social Functioning”. Finally, the last factor is comprised of subjective feelings of worry, sadness, stress, and anger, and thus the factor can be labeled as “Negative Affectivity”. Higher scores on the last factor would mean higher levels of Negative Affectivity.

### Replication of factor structure in data from 2007–2012

The same variables were constructed from GWP data collected between 2007 and 2012. The prosperity and globalization variables were related to 2007. As can be seen in Table 3, the emerging factor structure was largely similar to the results with more recent data. The same factors emerged. The 2007–2012 data included three extra variables for a smaller number of countries: happiness, life satisfaction these days, and purpose in life. These three variables were included in a separate factor analysis with a smaller sample size (*N* = 88). As shown in Table 4, the same factor structure was replicated. Life satisfaction these days loaded onto Socio-Economic Progress, and happiness and purpose in life loaded on Psycho-Social Functioning.

**Table 3:** Results of exploratory factor analysis (2007–2012, N = 141).

	Factor 1	Factor 2	Factor 3
Globalization	.**951**	-.067	.087
Economic quality	.**923**	.030	-.020
Education	.**909**	-.072	.038
Health	.**854**	.075	.107
Governance	.**852**	.106	-.061
Safety/security	.**850**	-.132	-.131
Business environment	.**843**	.152	-.053
Personal freedom	.**718**	.148	-.018
Life satisfaction (ladder)	.**710**	.372	.059
Natural environment	.**547**	.382	.005
Smile/laughter	.015	.**902**	.064
Eudaimonic well-being	.037	.**885**	.058
Well-rested	-.123	.**713**	-.175
Enjoyment	.197	.**664**	-.214
Future life satisfaction	.124	.**655**	-.125
Friendship opportunities	.281	.**553**	.007
Sadness	-.054	-.179	.**786**
Stress	.204	.232	.**695**
Worry	.076	-.197	.**681**
Anger	-.203	-.154	.**609**
Eigenvalues	8.948	3.273	1.523
% of Variance explained	44.740	16.366	7.614

*Note*. Factor loadings > .4 are shown in boldface.

**Table 4:** Results of exploratory factor analysis (2007–2012, N = 88).

	Factor 1	Factor 2	Factor 3
Globalization	.**942**	-.119	.040
Economic quality	.**923**	-.038	-.053
Education	.**902**	-.144	.057
Business environment	.**867**	.107	-.091
Health	.**860**	.048	.123
Governance	.**828**	.142	-.073
Life satisfaction (ladder)	.**798**	.289	.030
Safety/security	.**750**	-.132	-.193
Life satisfaction these days	.**707**	.303	.177
Personal freedom	.**670**	.205	.008
Natural environment	.**543**	.381	.096
Smile/laughter	.073	.**921**	.091
Happiness	-.031	.**897**	.045
Eudaimonic well-being	.002	.**852**	.014
Well-rested	-.056	.**765**	-.197
Purpose in life	-.**540**	.**667**	.045
Enjoyment	.259	.**648**	-.163
Future life satisfaction	.204	.**610**	-.171
Friendship opportunities	.307	.**571**	.028
Sadness	-.053	-.232	.**920**
Worry	.105	-.040	.**711**
Stress	.176	.242	.**650**
Anger	-.202	-.107	.**603**
Eigenvalues	8.988	4.333	2.252
% of Variance explained	39.076	18.838	9.791

*Note*. Factor loadings > .4 are shown in boldface.

### Factor correlations and country rankings

**Fig 1:**
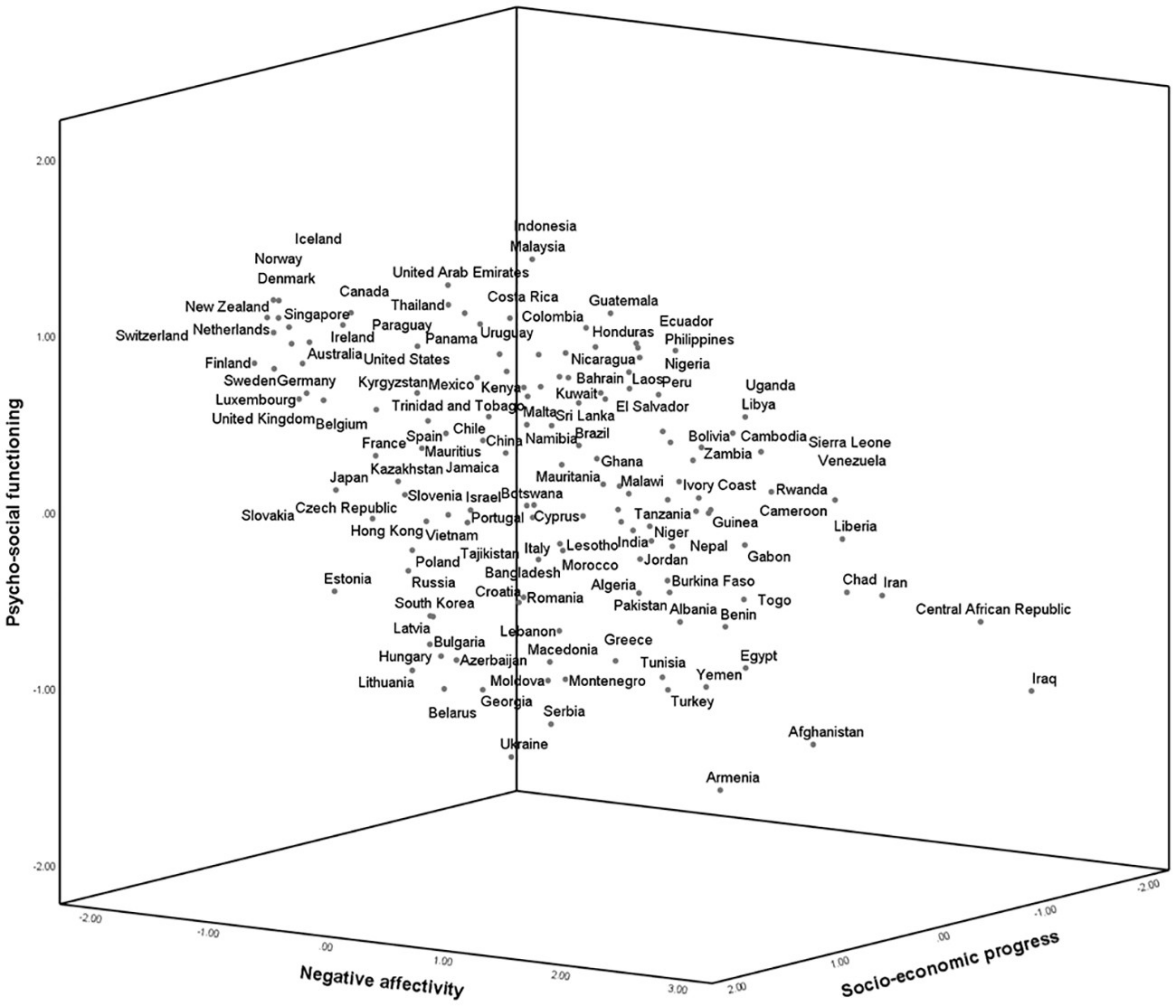
Three-dimensional scatterplot.

The consecutive analyses are entirely based on 2015–2017 data. The 20 variables (used in Table 2) were all z-standardized (to give a fairly equal presence to each variable in the final composite variables) and averaged to form three higher-level variables of Socio-Economic Progress, Psycho-Social Functioning, and Negative Affectivity. The Socio-Economic Progress score was calculated for nations with a single missing value as well. As shown in Table 5, observed correlations are stronger than latent correlations. In general, however, the correlations are modest, suggesting that a country’s standing on one of the dimensions does not strongly predict its standing on other dimensions. S1, S2 and S3 Figs in the supplementary material show the pairwise relationships between the variables. A three-dimensional scatterplot is shown in Fig 1. Cronbach’s alphas for each of the three variables based on the standardized variables are reported in Table 5, indicating acceptable internal consistency for each of the variables. Table 6 presents national scores and rankings.

**Table 5:** Factor correlations and alphas.

Factor	α	Latent variable correlations		Observed correlations	
		1	2	1	2
1. Socio-Economic Progress	.964	1.000		1.000	
2. Psycho-Social Functioning	.892	.338	1.000	.533	1.000
3. Negative Affectivity	.825	-.316	-.025	-.490	-.350

*Note*. The alphas are based on z-standardized variables. The observed correlation coefficients are all significant at *p* < .001

**Table 6:** Country scores.

	Socio-Economic Progress		Psycho-Social Functioning		Negative Affectivity	
*N*	149		152		152	
Country	score	rank	score	rank	score	rank
Afghanistan	-1.78	147	-1.66	149	0.63	30
Albania	-0.08	78	-0.69	123	0.99	18
Algeria	-0.42	101	-0.63	119	0.35	47
Angola	-1.38	142		.		.
Argentina	0.27	49	0.80	27	0.09	67
Armenia	-0.33	97	-1.67	150	1.11	13
Australia	1.54	10	0.99	15	-0.75	124
Austria	1.46	13	0.85	25	-0.88	133
Azerbaijan	-0.36	99	-1.12	139	-1.16	145
Bahrain	0.17	55	0.67	36	0.26	51
Bangladesh	-0.72	115	-0.50	112	-0.57	111
Belarus	-0.20	91	-1.26	143	-1.13	143
Belgium	1.39	15	0.62	41	-0.31	96
Belize	-0.14	87		.		.
Benin	-0.82	123	-0.85	127	0.72	28
Bhutan		.	0.79	28	-0.1	78
Bolivia	-0.11	84	0.41	56	1.41	9
Bosnia Herzegovina		.	-1.43	147	-0.16	83
Botswana	-0.11	82	-0.13	88	-0.28	94
Brazil	0.21	53	0.30	60	0.38	46
Bulgaria	0.22	51	-0.92	130	-0.88	132
Burkina Faso	-0.59	110	-0.57	117	0.44	41
Burundi	-1.19	140		.		.
Cambodia	-0.34	98	0.27	62	1.45	8
Cameroon	-0.80	120	-0.19	92	0.62	32
Canada	1.58	8	1.19	6	-0.36	101
Central African Republic	-1.80	149	-0.85	128	2.04	5
Chad	-1.56	143	-0.73	124	1.12	12
Chile	0.73	29	0.52	51	0.07	68
China	-0.09	79	0.20	67	-0.7	118
Colombia	0.03	65	0.94	18	0.29	50
Comoros	-1.05	136		.		.
Congo Brazzaville	-0.89	127	-0.38	111	-0.12	79
Congo Kinshasa	-1.66	145	-0.32	102	-0.24	91
Costa Rica	0.71	32	1.08	9	0.23	52
Croatia	0.51	43	-0.56	116	0.13	63
Cyprus	0.64	38	0.00	76	0.79	23
Czech Republic	0.98	23	-0.09	85	-0.7	117
Denmark	1.62	7	1.22	5	-0.99	138
Djibouti	-0.62	112		.		.
Dominican Republic	-0.02	73	0.54	48	0.41	44
Ecuador	0.13	58	0.90	21	0.8	22
Egypt	-0.59	111	-1.02	135	1.1	14
El Salvador	-0.01	70	0.61	42	0.62	31
Estonia	0.88	24	-0.55	114	-1.11	142
Ethiopia	-0.99	132	-0.29	99	-0.33	98
Finland	1.71	2	0.87	22	-1.07	141
France	1.34	17	0.35	57	-0.36	102
Gabon	-0.80	121	-0.37	109	0.91	20
Gambia		.	0.16	69	-0.15	82
Georgia	-0.12	85	-1.22	142	-0.73	121
Germany	1.56	9	0.70	35	-0.76	126
Ghana	-0.26	96	0.14	70	0.12	64
Greece	0.49	45	-0.83	126	0.94	19
Guatemala	-0.06	77	1.02	12	0.42	43
Guinea	-1.12	139	-0.28	98	0.32	48
Guyana	-0.23	94		.		.
Haiti		.	-1.77	151	0.55	36
Honduras	-0.23	92	0.78	30	0.14	60
Hong Kong	0.85	26	-0.09	86	-0.35	100
Hungary	0.47	46	-0.93	131	-0.56	109
Iceland	1.36	16	0.93	20	-1.06	140
India	-0.58	109	-0.36	107	0.31	49
Indonesia	-0.02	72	1.28	2	-0.21	90
Iran	-0.72	114	-0.54	113	2.15	3
Iraq	-1.60	144	-1.17	141	2.65	1
Ireland	1.40	14	1.08	10	-0.59	113
Israel	0.62	39	-0.05	81	-0.19	85
Italy	0.69	33	-0.27	95	0.46	40
Ivory Coast	-0.75	117	-0.04	79	0.39	45
Jamaica	0.17	56	0.20	66	-0.28	95
Japan	1.06	21	0.07	74	-0.95	136
Jordan	-0.10	80	-0.37	110	0.63	29
Kazakhstan	-0.01	71	-0.07	82	-1.35	149
Kenya	-0.51	105	0.44	54	-0.72	119
Kosovo		.	-0.12	87	-0.88	131
Kuwait	0.11	59	0.59	44	0.48	39
Kyrgyzstan	-0.10	81	0.42	55	-1.27	147
Laos	-0.54	107	0.59	45	0.15	59
Latvia	0.65	36	-0.67	121	-0.48	107
Lebanon	-0.26	95	-0.86	129	-0.2	86
Lesotho	-0.86	125	-0.33	104	-0.2	87
Liberia	-1.06	137	-0.32	103	1.51	7
Libya	-0.98	131	0.32	58	0.76	24
Lithuania	0.57	41	-1.01	134	-0.72	120
Luxembourg	1.52	12	0.65	38	-0.86	129
Macedonia	0.22	52	-0.94	133	0.14	62
Madagascar	-1.00	133	0.12	71	0.1	66
Malawi	-0.72	116	-0.14	89	-0.01	75
Malaysia	0.64	37	0.70	34	0.43	42
Mali	-1.04	134	0.17	68	0	74
Malta	0.87	25	0.53	49	0.51	37
Mauritania	-1.31	141	-0.23	93	-0.74	122
Mauritius	0.62	40	0.27	64	-0.6	114
Mexico	0.23	50	0.62	40	-0.47	106
Moldova	-0.23	93	-1.15	140	-0.27	93
Mongolia	-0.13	86	0.27	63	-1.2	146
Montenegro	0.08	61	-1.06	138	0.16	58
Morocco	-0.17	89	-0.35	106	-0.12	80
Mozambique	-0.87	126	-0.18	91	0.19	56
Myanmar		.	0.29	61	-0.06	76
Namibia	-0.17	88	0.31	59	-0.18	84
Nepal	-0.45	103	-0.35	105	0.6	34
Netherlands	1.62	6	1.12	7	-0.94	135
New Zealand	1.65	4	1.12	8	-1.01	139
Nicaragua	0.03	66	0.86	24	0.72	27
Niger	-1.12	138	-0.27	97	0.22	53
Nigeria	-0.97	130	0.59	46	-0.14	81
Northern Cyprus		.	-0.17	90	0.61	33
Norway	1.71	3	1.24	3	-0.86	130
Oman	0.06	64		.		.
Pakistan	-1.04	135	-0.74	125	0.06	70
Palestine		.	-0.93	132	1.16	10
Panama	0.51	44	0.99	16	-0.2	88
Paraguay	0.02	67	0.93	19	-0.76	125
Peru	0.13	57	0.63	39	0.99	17
Philippines	-0.01	69	0.86	23	1.01	16
Poland	0.73	30	-0.29	100	-0.58	112
Portugal	0.84	27	0.06	75	0.49	38
Qatar	0.54	42		.		.
Romania	0.39	47	-0.56	115	0.06	69
Russia	-0.11	83	-0.59	118	-1.36	150
Rwanda	-0.39	100	-0.03	77	0.2	55
Saudi Arabia	0.02	68	0.78	31	0.1	65
Senegal	-0.53	106	0.52	50	-0.43	105
Serbia	0.07	63	-1.33	145	0.02	72
Sierra Leone	-0.93	128	-0.07	83	1.57	6
Singapore	1.30	18	1.01	13	-1.13	144
Slovakia	0.69	34	-0.08	84	-0.31	97
Slovenia	1.04	22	0.08	73	-0.37	103
Somalia		.	0.76	32	-1.34	148
South Africa	0.10	60	0.74	33	-0.39	104
South Korea	0.67	35	-0.67	120	-0.48	108
South Sudan		.	-1.32	144	2.07	4
Spain	1.09	20	0.47	53	0.02	73
Sri Lanka	-0.02	74	0.49	52	0.18	57
Sudan	-1.79	148		.		.
Suriname	-0.03	76		.		.
Swaziland	-0.94	129		.		.
Sweden	1.64	5	0.84	26	-0.96	137
Switzerland	1.73	1	1.06	11	-0.89	134
Syria		.	-3.29	152	2.41	2
Taiwan		.	0.59	43	-1.44	151
Tajikistan	-0.42	102	-0.27	96	-0.56	110
Tanzania	-0.76	118	-0.31	101	0.14	61
Thailand	0.19	54	1.01	14	-0.75	123
Togo	-0.81	122	-0.68	122	0.89	21
Trinidad and Tobago	0.32	48	0.57	47	0.04	71
Tunisia	-0.19	90	-1.04	136	0.74	26
Turkey	0.08	62	-1.05	137	1.03	15
Turkmenistan		.	-0.37	108	-0.33	99
Uganda	-0.78	119	-0.04	78	1.16	11
Ukraine	-0.46	104	-1.65	148	-0.78	128
United Arab Emirates	0.76	28	1.24	4	-0.26	92
United Kingdom	1.54	11	0.67	37	-0.63	115
United States	1.26	19	0.98	17	-0.08	77
Uruguay	0.71	31	0.78	29	0.2	54
Uzbekistan		.	1.56	1	-1.5	152
Venezuela	-0.69	113	0.10	72	0.57	35
Vietnam	-0.02	75	-0.26	94	-0.77	127
Yemen	-1.70	146	-1.39	146	-0.21	89
Zambia	-0.57	108	0.21	65	0.74	25
Zimbabwe	-0.83	124	-0.04	80	-0.68	116

### Regional distribution of national prosperity and well-being

**Fig 2:**
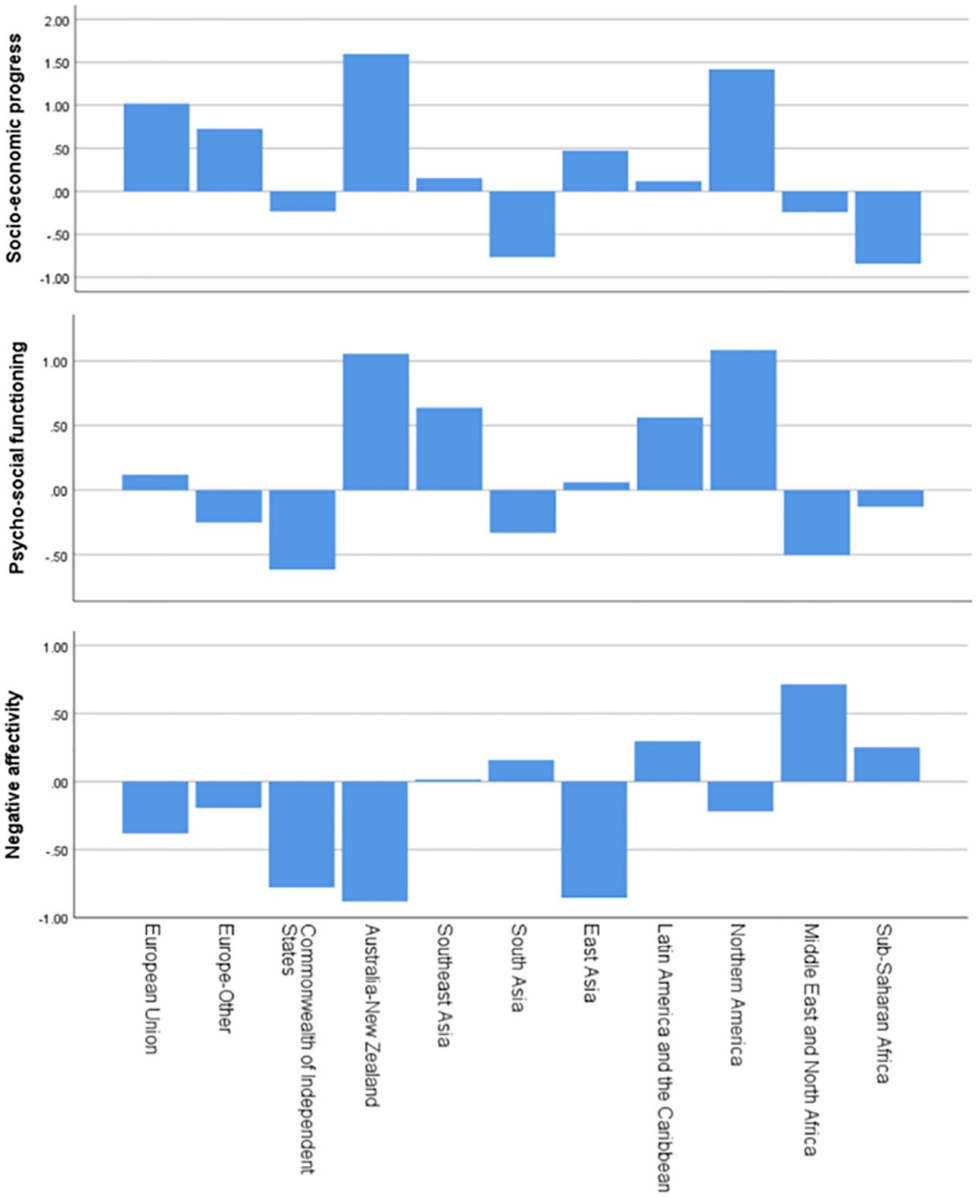
Regional averages.

Fig 2 shows the regional levels of the three prosperity variables. The names of countries in each category are shown in S4 Table. In terms of Socio-Economic Progress, Northern America, Australia, and New Zealand have the highest scores, whereas Sub-Saharan Africa and South Asia have the lowest scores. On Psycho-Social Functioning, Northern America, Australia, and New Zealand have the highest scores; Commonwealth of Independent States and Middle East and North Africa have the lowest scores. Finally, on Negative Affectivity, Middle East and North Africa have the highest score, whereas Commonwealth of Independent States, Australia, and New Zealand and East Asia have the lowest scores. Note that a high score on Negative Affectivity is not a ‘good’ score–high levels of negative affect are undesirable. The top-ranked countries here are Iraq and Syria, both blighted by civil war in recent years.

### The nomological network of the three dimensions

Table 7 presents the correlations between the three prosperity variables and the external variables of the study. The correlations with the four variables removed from the factor analysis are also presented in the table. As can be seen, most of the variables have differential associations with the external variables. This suggests that the variables have partly unique nomological networks.

**Table 7:** Correlations between the well-being dimensions and external variables.

	Income inequality	Anxiety	Depression	Suicide	Religiosity	Power distance	Individualism
Socio-Economic Progress	-.258**	.559***	.389***	-.078	-.722***	-.609***	.687***
*N*	136	148	148	148	148	68	68
Psycho-Social Functioning	.196*	.253**	-.032	-.022	-.120	-.265*	.128
*N*	137	149	149	147	152	68	68
Negative Affectivity	.254**	-.005	-.146	-.195*	.536***	.351**	-.359**
*N*	137	149	149	147	152	68	68
	Uncertainty avoidance	Secular values	Self-expressive values	Cold demands	Heat demands	% Urban	% Aged over 65
Socio-Economic Progress	-.205	.563***	.716***	.500***	-.442***	.642***	.780***
*N*	68	87	87	149	149	149	149
Psycho-Social Functioning	-.303*	.015	.775***	-.120	-.079	.292***	.066
*N*	68	90	90	148	148	150	148
Negative Affectivity	.317**	-.573***	-.235*	-.442***	.293***	-.187*	-.384***
*N*	68	90	90	148	148	150	148

*Note*. Masculinity was not significantly correlated with any of the dimensions and thus is not presented in the table. *N* = sample size.

*p < .05;

**p < .01;

***p < .001

## Discussion

Based on the results of the factor analysis, we identified three interpretable factors suggesting that, within the complex picture presented by the wide range of available indicators, distinct concepts are discernible. Below, we elaborate on the nature of the three factors.

### Factor 1: Socio-Economic progress

This factor is loaded by variables that mostly reflect external conditions of life (as opposed to subjective and psychological feelings), country-level situations, and the functioning of public, social, and political institutions. Globalization and all of the prosperity sub-indices loaded on this factor. The indicators of this factor are partially based on people’s opinions as expressed in large-scale surveys, with survey questions mostly capturing people’s opinions about the socio-economic situations around them. The variables loading on this factor are primarily dependent on economic growth. Surprisingly, life satisfaction (ladder of life) too loaded on this factor, as its only purely subjective indicator. Cantril’s ladder of life has been shown to be more closely associated with socio-economic indicators than standard single-item life satisfaction measures (e.g., All things considered, how satisfied are you with your life as a whole these days?) [50]. However, in our supplementary analysis, we found that satisfaction these days also loaded on Factor 1 (Table 4). Unlike other subjective indices (such as affect and eudaimonic well-being), national life satisfaction does not seem to reflect well how life is subjectively experienced on a day-to-day basis. Instead, it seems largely to reflect how people perceive, or are affected by, economic and socio-political developments in their nations. This finding is consistent with evidence that economic, political, and social stress at the national level (e.g., crisis and war) can lead to rather rapid declines in a nation’s level of life satisfaction [40, 79, 80].

Life satisfaction can be seen as an indicator of the extent to which people are getting what they want in life. Our results suggest that, when people’s satisfaction responses are aggregated to calculate national scores of satisfaction, they align well with countries’ objective levels of development. Previous research has also found strong correlations (e.g., > .7) between national life satisfaction and economic and prosperity indicators [33, 40, 81]. Thus, it can be surmised that life satisfaction questions (particularly ladder of life) encourage people to think of their lives in economic terms [82]. This raises the question whether the overlap between country-level life satisfaction and Socio-Economic Progress is sufficient to create redundancy such that the simultaneous use of socio-economic indicators and life satisfaction at the country level adds little benefit, or worse, causes issues of multicollinearity (e.g., when using these indicators simultaneously as predictors in regression models). This large overlap also suggests that the availability of reliable measures of socio-economic development for a country may reduce the need for relying on life satisfaction measures, if the outcome variable of interest is Socio-Economic Progress. Conversely, however, given its strong loading on the first factor, national life satisfaction could be considered as a valid and highly useful summary index of general socio-economic development in large groups (e.g., nations). This is particularly so at the community or group level (e.g., with ethnic and cultural groups within nations, socio-economic classes, and neighborhoods), where reliable group-level indicators may be nonexistent.

### Factor 2: Psycho-Social functioning

This factor is more subjective than Factor 1, being loaded by positively-valenced mental states (smile/laughter, future life satisfaction, well-rested, and enjoyment) as well as eudaimonic well-being and friendship opportunities. In the additional analysis, we found that happiness (close to enjoyment) and purpose in life (close to eudaimonic well-being) also loaded on this factor. These variables have in common an emphasis on inner feelings, psychological and interpersonal resources and functioning in life rather than external living conditions. This factor also covers social domains of well-being including altruism and social capital. In sum, this factor reflects a version of well-being that emphasizes living a worthwhile, hopeful, and enjoyable life involving pleasant states of mind but also developing and exercising personal and social skills.

Our finding that life satisfaction loaded on Factor 1 but affective variables loaded on other factors resonates with previous research concerning differential results for life satisfaction and positive affect. Earlier studies have found that national happiness is not correlated with life-satisfaction [83], and that affective measures are more sensitive to circumstances that evoke positive and negative emotions and less so to socioeconomic status, economic conditions, income, and material variables [84–87]. The fact that life satisfaction measures loaded on Factor 1 and happiness loaded on Factor 2 suggests that different conclusions can be drawn using different SWB measures, and that findings produced by one measure might not hold for other measures. Thus great caution is needed when comparing the results of studies using different measures of well-being [88].

An important finding is that the present and future life satisfaction loaded on different factors. Gallagher, Lopez, and Pressman [89] argue that future life satisfaction measures the expectation of positive future outcomes and thus is a face-valid measure of optimistic expectations for the future. At the individual level, future life satisfaction has been found to have a stronger correlation with optimism and hope than do present and past satisfaction [90, 91]. The present country-level results are in keeping with the previous results. Our results show that, compared with present life satisfaction, future life satisfaction is associated less strongly with current socio-economic conditions and more strongly with positive affect and eudaimonic well-being. These results suggest that, at the country level, future life satisfaction is more likely to measure positive emotions and attitudes such as hope and optimism than evaluations of objective living conditions. Given the dearth of country-level studies involving future life satisfaction, these explanations and suggestions need to be further investigated in future studies. Yet, our findings do clearly indicate that future and present life satisfaction are distinguishable in interpretable ways, which needs to be acknowledged in future research.

We found that eudaimonic well-being also did not load on socio-economic prosperity. This too is consistent with previous research [33, 92]. The present results and previous research [93] leads us to predict that eudaimonic well-being is less likely than life satisfaction to change rapidly in response to economic and political stress at the national level, a prediction to be tested empirically in future research.

### Factor 3: Negative affectivity

Of the three, Factor 3 is the most homogeneous: sadness, worry, stress, and anger, are all negatively-valenced affective states, and can be broadly categorized as forms of displeasure. Hence, this factor captures individuals’ day-to-day experience of unpleasant affect, and thus can be considered a measure of ill-being. However, it can be transformed into a positive well-being measure if reverse-scored. Whereas the separation of Factor 1 from the other two factors is interpretable in view of the level of subjectivity of the indicators concerned, the distinction between Factors 2 and 3 is not based on the levels of subjectivity but on the valence of the subjective states. This distinction is consistent with the predominant view that positive and negative affect are largely independent of each other, rather than two sides of the same coin [94]. Grinde [51] argues that this distinction is particularly important in that positive and negative affect have differential associations with one’s overall level of functioning.

Helliwell et al. [55] showed that positive emotions were more strongly associated with life satisfaction than were negative emotions and that positive and negative affect had differential relationships with other variables (e.g. generosity and perceptions of corruption). Our results indicate that, at the country level, positive affect is more strongly associated with eudaimonic well-being, future satisfaction, happiness, friendship opportunities, and feeling well-rested than is negative affect. Our results converge with the previous results in suggesting that positive and negative affect capture two different aspects of country-level well-being and should be studied independently.

### Nomological networks

The three factors showed clearly distinct nomological networks. Below, we highlight some of the findings related to each of the factors.

#### Factor 1: Socio-Economic progress

This dimension was correlated with all of the external variables, except suicide and uncertainty avoidance. The strongest correlations were with national age (i.e., % aged over 65), religiosity (negative), self-expressive values, and individualism. This pattern is consistent with the general expectations of socio-economic development. For example, developed countries tend to have older populations, and endorse more secular and individualistic values [95]. The factor has a positive correlation with cold demands and a negative association with heat demands. It is also correlated with anxiety and depression. People in richer countries are less likely to report that their lives have a meaning [92], which may contribute to higher levels of depression. Research has also found that richer countries are also more likely to have a higher prevalence of some health problems such as obesity, and alcohol use [96–98]. Therefore, a high level of Socio-Economic Progress is not a panacea, and these countries still have challenges to face. It is not clear whether these challenges are products of higher Socio-Economic Progress or remnants of pre-existing problems that Socio-Economic Progress has not yet been able to solve; or whether rising levels of Socio-Economic Progress will resolve these issues, intensify them, or leave them unaffected.

#### Factor 2: Psycho-Social functioning

This factor had relatively weaker correlations with the external variables than the other two factors, which reflects its strongly subjective content. It has its strongest correlation with self-expressive values, other correlations being nonsignificant, weak, or moderate. Unlike the other two factors, it had no association with temperature. This pattern of associations suggests that societies with higher levels of Psycho-Social Functioning are more likely to emphasize subjective quality of life and self-expression over economic and physical security [95], and are open to situations that are novel, unknown, and different from usual [75]. We speculate that Psycho-Social Functioning’s orthogonality to suicide as reflected in the multidimensional plots is largely a result of its association with meaning in life. A strong endorsement of self-expressive values is one of the main commonalities between countries that are high both on Socio-Economic Progress and Psycho-Social Functioning. Finally, Psycho-Social Functioning has a weak but significant positive correlation with anxiety.

Psycho-Social Functioning’s weaker associations with secular/individualistic values as well as economic and political situations suggests that developing countries are more likely to score well on this type of well-being than on Socio-Economic Progress. This is reflected in the fact that some African countries which suffer from life dissatisfaction have better standings on eudaimonic well-being (e.g., Mali, Libya, Nigeria).

#### Factor 3: Negative affectivity

This variable has weaker associations with the external variables than Socio-Economic Progress. Its strongest correlation is with secular values (negative), religiosity (positive), and cold demands (negative). The pattern of correlations for this variable suggests that negative affect is higher in more collectivistic, hotter, less urbanized countries, countries with younger populations and higher levels of uncertainty avoidance, power distance, and income inequality. This suggests that negative affect is inversely associated with socio-economic development and the endorsement of progressive values.

### Dimensionality of national quality of life

Our data-driven exploratory analysis provides a plausible framework for understanding and appraising quality of life at societal level, and supports the view that country-level quality of life is a multidimensional construct, not a holistic entity that can be measured adequately by a single measure. Our two-dimensional plots of variables do not provide evidence of centrality for any of the variables. Our results are consistent with Diener and Suh’s [13] conclusion that objective, and subjective indicators are complementary, each capturing different aspects of societal well-being, and thus should be used in parallel. Therefore, it is necessary for future research to study the three dimensions separately as well as in tandem.

Quality of life of a given country differs substantially depending on the measure used to operationalize it. For example, the use of objective measures of material wealth may produce different rankings from measures of social and psychological resources [56, 99]. Similar findings were observed in the present study. All the countries that ranked in the top ten on the Socio-Economic Progress dimension (Switzerland, Finland, Norway, New Zealand, Sweden, the Netherlands, Denmark, Canada, Germany, Australia) are affluent countries from Nordic, Anglo, and Western-European societies. On the other hand, the top 10 countries in the psychosocial functioning dimension are more diverse (Uzbekistan, Indonesia, Norway, United Arab Emirates, Denmark, Canada, the Netherlands, New Zealand, Costa Rica, Ireland), as are the 10 countries that rank lowest in Negative Affectivity (Uzbekistan, Taiwan, Russia, Kazakhstan, Somalia, Kyrgyzstan, Mongolia, Azerbaijan, Singapore, Belarus).

Our regional analysis also shows the importance of considering the dimensions separately. Whilst the pattern of high and low scores across different regions was broadly similar between Socio-Economic Progress and Psycho-Social Functioning, South and Southeast Asia, Latin America and Sub-Saharan Africa scored higher on the latter than the former, whereas the reverse was true of European, Commonwealth of Independent States and Middle Eastern countries. Comparing Negative Affectivity with the other two factors revealed some more marked differences. Commonwealth of Independent States and East Asian countries had much better scores on this factor than the other two (a low score on Negative Affect being a good score); Northern America rather worse. These differences underline the complexity of prosperity and well-being at the national level. The fact that certain countries scored better or worse on Socio-Economic Progress than the other factors suggests that the aspects of prosperity which make up this factor do not tell the whole story of what influences well-being at the national level. Some countries, such as Nigeria and Paraguay, scored markedly better on Factors 2 and 3 than Factor 1 (Table 6). Therefore, a nation’s standing on one dimension may not accurately reveal its standing on the others. These findings militate against a reductive and simplistic approach that assumes a single gold standard measure of quality of life. Our results unmistakably indicate that using a single measure of national prosperity is inappropriate and may lead to simplistic conclusions.

Although positive and negative affect along with life satisfaction tend to form a SWB factor in studies on individual-level indicators of well-being (for a review, see [39]), in our national level analysis, these variables loaded on three distinct factors. Thus, conclusions about the structure of well-being obtained at the individual level should not be automatically translated to assumptions about the structure of prosperity and well-being at the national level.

An interesting finding was that Socio-Economic Progress and Psycho-Social Functioning had a stronger correlation than Psycho-Social Functioning and Negative Affectivity (Table 5). In general, the Negative Affectivity factor is more distinct from the other two factors. This finding is difficult to explain given that negative affect has been rarely studied at national level and not much is known about it. Yet, the finding is consistent with Helliwell et al.’s [55] findings suggesting that negative affect is less fully explained by other well-being and prosperity variables than are life satisfaction and positive affect, and is less strongly associated with life satisfaction than positive affect is. In sum, these results are suggestive of both complementarity of and overlap between the three dimensions.

### Anxiety, depression, suicide, and income inequality

The four variables of anxiety, depression, suicide, and income inequality are considered by some as important components of country-level quality of life [28, 52]. For anxiety, depression, and suicide, these assumptions seem to be largely based on individual-level findings. For example, a depressed and anxious person is almost certainly not very happy. Yet, we found that, at the national level, these variables do not connect with the other indicators in an expected manner, which may cast doubt on their utility in measuring national quality of life (i.e., quality of life in the whole national population).

Depression had a positive loading on Factor 1, and its loading on Factor 3 was almost zero. Yet, it had a weak negative loading on Factor 2, which is consistent with the general expectations, since this factor involves variables such as meaning in life and positive affect that are antithetical to depression. Anxiety also had an expectedly positive loading on the first factor, and a loading in the expected direction but weaker on Factor 3. Given these mixed results, it is doubtful that depression and anxiety can be used as unambiguous indicators of national quality of life. Instead, we suggest using depression and anxiety as indicators of quality in life in depressed and anxious populations in a given country, as vulnerable populations that deserve special care. It is worth noting that we used the DALYs due to anxiety and depression (produced by the global burden of disease study), which are incidence-based measures that combine both mortality and morbidity into a single value [73]. Alternative national measures of anxiety and depression (e.g., prevalence-based measures) may lead to different conclusions.

Surprisingly, suicide had a weak but nontrivial negative loading on Factor 3, and trivial loadings on the other factors. Some previous studies have also found positive correlations between suicide rates and SWB [100, 101]. The suicide rate has been excluded from the most recent update of the Social Progress Index due to low correlations with other indicators of health and wellness [102]. Daly et al. [100] suggest that personal unhappiness may be at its worst when surrounded by those who are relatively more content with their lives. Diener and Suh [13] suggest that in high-SWB and high-income countries, there may be less social support and security when life goes badly as well as higher chances of loneliness which may lead to higher rates of suicide. In other words, higher levels of freedom in these countries have both the desirable effect of increasing SWB and the undesirable effect of increasing the possibility of suicide for people whose lives go badly. Based on these findings, national suicide rates cannot be used as an unambiguous nation-level quality of life indicator, and instead could be used for monitoring the status of people with suicidal tendencies and other affected populations in any given country.

Income inequality has shown positive, negative, and ambiguous associations with SWB [40, 103]. There is evidence that inequality may increase well-being in some nations but not others [104–106]. Similarly, inequality’s association with economic growth is also not clear-cut [107]. We also found mixed results for income inequality. It had a negative loading on the first factor, and surprisingly a positive loading on the second factor. Therefore, our results dovetail with the previous evidence in suggesting that income inequality is not an unambiguous indicator of national quality of life. Therefore, we suggest not including income inequality as an indicator of country-level quality of life. Instead, income inequality could be studied in its own right as an important nation-level variable that can have complex and heterogeneous relationships with various aspect of national quality of life in various contexts and groups.

In view of the mixed pattern of associations with the other indicators, we excluded these variables from our factor analysis and instead used them as external variables to establish the nomological networks of the quality of life variables. We acknowledge that theory-driven treatments of national quality of life may be more open to including these four variables as component parts, yet, our data-driven approach hinders this on the grounds of the ambiguous factor analytic and correlational findings for these variables.

### Relevance to public policy

This paper was intended to investigate holistically the structure of prosperity and well-being at the national level rather than to directly inform public policy. Nevertheless, we do believe that our findings would be of interest to policy makers. For example, the fact that our results support a broadly-based approach to measuring well-being, involving a mix of subjective and objective measures and avoiding over-reliance on any single type of measure is relevant to national well-being programmes as well as to academic research. In addition, the performance of nations varies across the three factors, so that a nation’s standing on one dimension does not reveal its standing on the others. This clearly cautions against over-reliance on unidimensional means of improving well-being–for example, an exclusive focus on economic prosperity. Thus, our results provide a comprehensive and multidimensional picture of well-being to inform the development of public policy. We hope that future studies will build on our work to provide results of more direct relevance to specific policy issues. For example, future studies might consider factor-analysis on sub-categories of measures organized by different policy objectives rather than across the full range.

### Limitations and future research

Some of the limitations of this study need to be mentioned. First, the choice of indicators affects the results of factor analysis. The obtained factor structure in any factor analysis is inevitably contingent on the selected variables. Our selection of variables of national well-being is of course not definitive. Other subjective and objective indicators of national well-being could be incorporated in future studies on the structure of human well-being at the national level, to see if conclusions are affected in significant ways. As new scientific methods and data sources for measuring well-being at the nation level are emerging (for example, the use of language or text data to infer national well-being [108]), future studies can include more measures of well-being from diverse sources. Second, the GWP has its limitations, e.g., the single-item measures used to assess most of the concepts. Future research will need to use more reliable measures. Subjective measures of quality of life have been criticized on various grounds, including equivalence in translation and psychometrics [109, 110]. For example, researchers have emphasized that to make meaningful cross-cultural comparisons, equivalence and bias need to be addressed in cross-cultural surveys [111, 112]. More research in this area is needed to examine the magnitude of the effect of these artifacts on the measurement of well-being across cultures. Third, we used a limited number of external variables to validate the structure of national well-being that emerged in the present study. Future studies could include additional predictors or outcomes of well-being such as progressive taxation, personality traits, and crime rates (for a review, see [58]). Fourth, given the cross-sectional nature of our data, causality may not be inferred and future research needs to use longitudinal research designs to shed light on the direction of causality.

Finally, cross-national comparisons and ranking of countries are subject to a variety of caveats, which deserve special attention. The ranking of nations against the three factors: Factor 3 in particular–produced some surprising results. For example, whilst Iraq and Syria, which have endured bloody internal conflict, had the highest levels of negative affect, Somalia, a country experiencing similar strife, had one of the lowest. The predominance of former Soviet countries among those reporting the lowest levels of negative affect is also difficult to explain. We acknowledge that these–and perhaps other–results may have been influenced by other factors affecting the responses (e.g., for example, cultural factors or response sets) as well as by actual levels of negative affect. As argued by Tov and Au [113] cross-national differences in well-being and country ranking on well-being variables need to be interpreted with caution. The equivalence of meaning of SWB concepts (e.g., life satisfaction and happiness) should be carefully inspected in studies aimed at comparing countries on well-being measures, because these terms might mean different things to individuals from different cultures. For example, previous studies have found that meanings of, and attitudes to, terms such as “happiness” and “life satisfaction” may vary across languages and cultures [110, 114–116]. Therefore, a necessary avenue for future research would be to tease out cultural influences systematically.

### Concluding remarks

The limitations of GDP and similar wealth indicators have led to the development of numerous ideas and frameworks to measure national quality of life and provide information relevant to public policy. SWB, particularly its cognitive component (i.e., life satisfaction) has received extensive attention in this area of research and practice. Our results expanded these insights in various ways. For example, we found that, in comparison to life satisfaction, the affective components of SWB reflect information that is more likely to be different from that already captured by economic indicators. Moreover, our results on the structure of prosperity and well-being variables provide a systematic way to look at the diverse indicators and their interrelations. For example, the results suggest that eudaimonic well-being has stronger associations with positive affect than life satisfaction and Socio-Economic Progress, and eudaimonic well-being is also more likely than life satisfaction to provide information that is different from economic indicators. Our results also call into question the practice of categorizing affect and life satisfaction under the title of “subjective well-being” at the country level. This conceptualization stems from individual-level results and we showed in this article that it was not consistent with country-level results.

Delle Fave [30] highlights the current need for a more integrated and articulated perspective in the selection, use and interpretation of well-being indicators. One way to make progress towards satisfying this pressing need is data-driven explorations of the factor structure of the societal indicators of quality of life. Our study seems to be the first broad and inclusive study of this sort, aimed at organizing a large list of indicators in a conceptual structure. Inquiry into the structure of well-being at the national level is of crucial importance at this stage of development in this field of research. The results of such studies can help us avoid over-reductionism, and instead enable a systematic identification of elementary indictors to efficiently reduce the number of variables. Such studies help to balance the hegemony of particular approaches over others [117]. They can also support the development of empirically-informed conceptual frameworks. It is, however, appropriate to be cautious at this stage, in view of the fact that this study is among the first factor-analytic studies of prosperity and well-being at the country level. Thus, our findings should be considered preliminary. It is hoped that these results will stimulate more research endeavors aimed at clarifying the structure of national prosperity and well-being. It is noteworthy that studies of this nature involve ecological analyses the results of which do not necessarily have implications for individuals [83].

In conclusion, the results of our research paint a complex and promising–if provisional–picture of prosperity and well-being at the national level. The complexity of the relationships between the three well-being factors, and between them and other variables, underlines the need for a broadly-based approach to the measurement of well-being, avoiding over-reliance on any single indicator such as life satisfaction. We hope that other researchers will carry out further work to expand and advance these findings.

## Supporting information

S1 Fig The relationship between psycho-social functioning and socio-economic progress. (DOCX)

S2 Fig The relationship between negative affectivity and socio-economic progress. (DOCX)

S3 Fig The relationship between negative affectivity and psycho-social functioning. (DOCX)

S1 Table Descriptive information for the main dataset (2015–2017). (DOCX)

S2 Table Gallup items used in the study. (DOCX)

S3 Table Intercorrelations between the 24 indicators. (XLSX)

S4 Table Global regions (Gallup’s categorization). (DOCX)
