# Supplementary figures and images for: A multidimensional understanding of prosperity and well-being at country level: Data-driven explorations
Source: PLoS One. 2019 Oct 9;14(10):e0223221. doi: 10.1371/journal.pone.0223221 (PMC6785080; doi:10.1371/journal.pone.0223221)

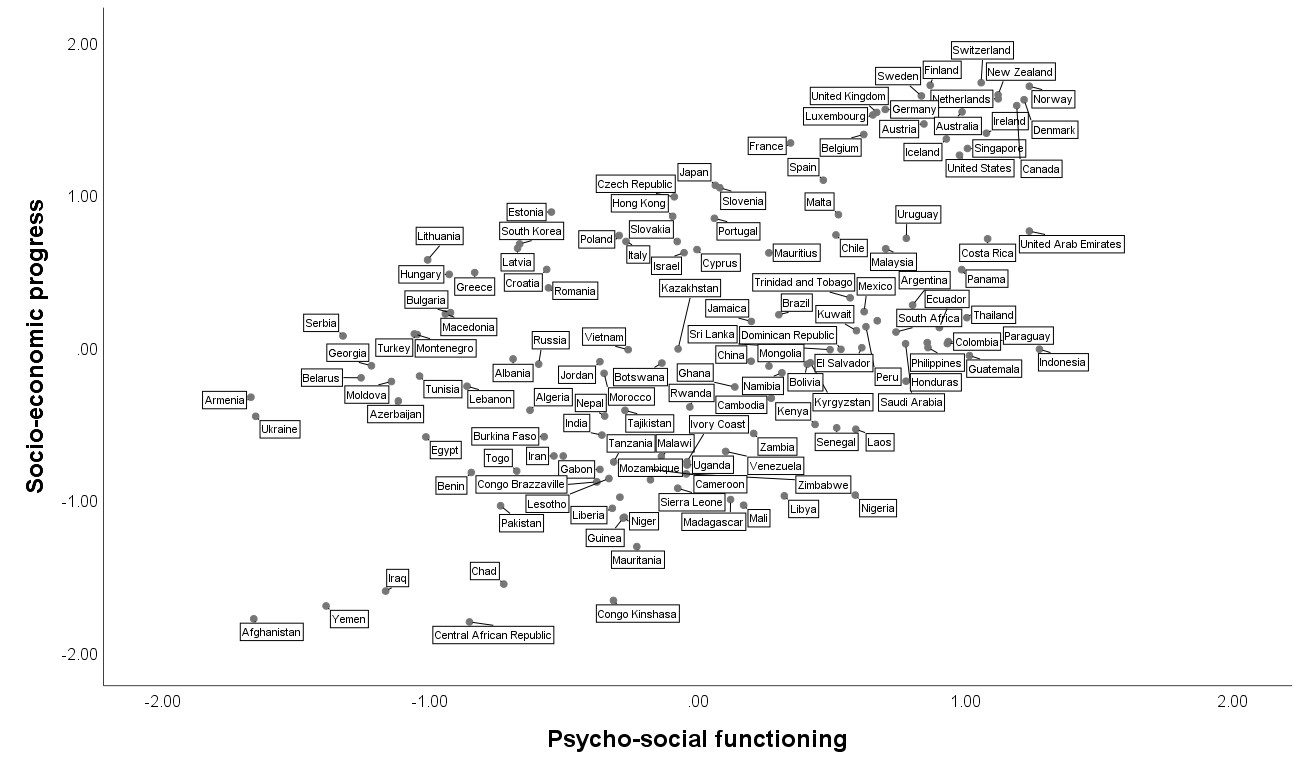


Figure S1

The relationship between psycho-social functioning and socio-economic progress

Supplement: S1 Fig — (DOCX) [file pone.0223221.s001.docx]

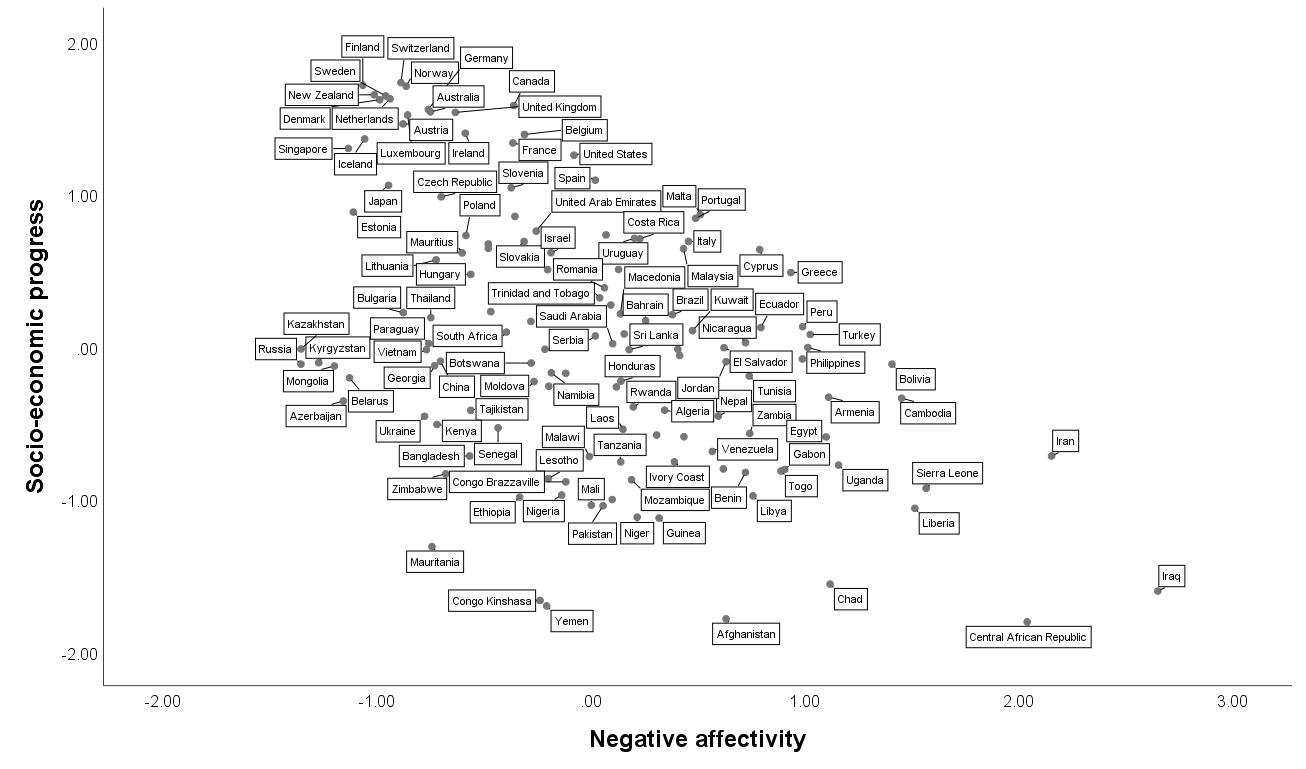


Figure S2

The relationship between negative affectivity and socio-economic progress

Supplement: S2 Fig — (DOCX) [file pone.0223221.s002.docx]

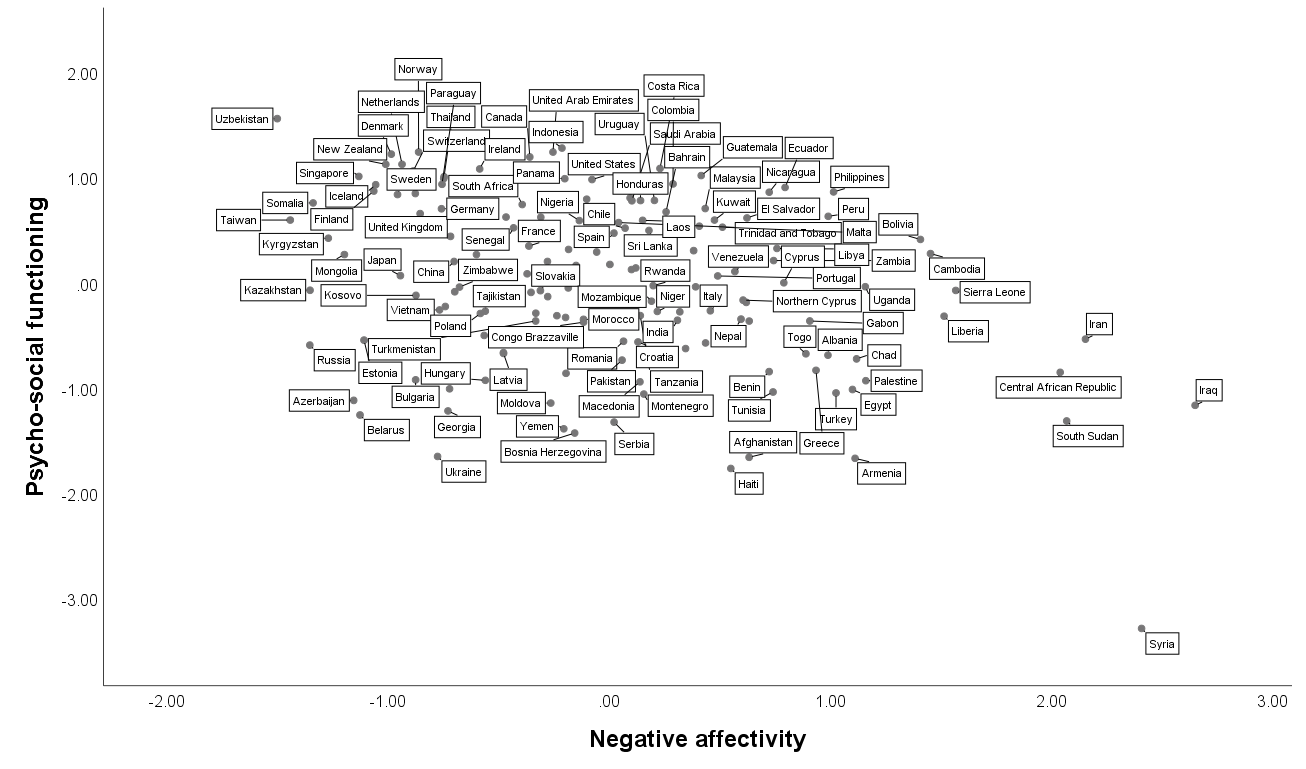


Figure S3

The relationship between negative affectivity and psycho-social functioning

Supplement: S3 Fig — (DOCX) [file pone.0223221.s003.docx]
