# Supplementary material for: A multidimensional understanding of prosperity and well-being at country level: Data-driven explorations
Source: PLoS One. 2019 Oct 9;14(10):e0223221. doi: 10.1371/journal.pone.0223221 (PMC6785080; doi:10.1371/journal.pone.0223221)
Supplement: S1 Table — (DOCX) [file pone.0223221.s004.docx]

| **Table S1**  ***Descriptive Information for the Main Dataset (2015-2017)*** | | | |
| --- | --- | --- | --- |
|  | *N* | Age | Female % |
| Afghanistan | 3000 | 33.59 | 49.9% |
| Albania | 2999 | 45.93 | 60.2% |
| Algeria | 2017 | 37.88 | 50.9% |
| Argentina | 3000 | 45.55 | 59.1% |
| Armenia | 3000 | 46.77 | 58.0% |
| Australia | 3008 | 58.94 | 57.4% |
| Austria | 3000 | 46.74 | 51.7% |
| Azerbaijan | 3000 | 38.22 | 51.7% |
| Bahrain | 4078 | 36.04 | 34.9% |
| Bangladesh | 3000 | 36.74 | 52.9% |
| Belarus | 3126 | 44.61 | 59.5% |
| Belgium | 3038 | 48.56 | 47.3% |
| Benin | 3000 | 32.30 | 47.0% |
| Bhutan | 1020 | 38.13 | 48.1% |
| Bolivia | 3000 | 38.63 | 58.7% |
| Bosnia Herzegovina | 3000 | 48.66 | 54.4% |
| Botswana | 3000 | 38.26 | 66.0% |
| Brazil | 3005 | 44.67 | 59.4% |
| Bulgaria | 3000 | 53.44 | 58.3% |
| Burkina Faso | 3000 | 32.29 | 40.1% |
| Cambodia | 3600 | 40.67 | 66.3% |
| Cameroon | 3000 | 32.61 | 52.4% |
| Canada | 3032 | 52.78 | 51.6% |
| Central African Republic | 2000 | 32.77 | 51.9% |
| Chad | 3000 | 32.50 | 42.3% |
| Chile | 3088 | 46.39 | 61.3% |
| China | 12779 | 46.99 | 52.9% |
| Colombia | 3000 | 42.66 | 61.3% |
| Congo Brazzaville | 3000 | 35.55 | 51.2% |
| Congo Kinshasa | 3000 | 31.93 | 42.4% |
| Costa Rica | 3000 | 45.45 | 58.6% |
| Croatia | 3000 | 44.77 | 54.3% |
| Cyprus | 3043 | 46.04 | 51.7% |
| Czech Republic | 3000 | 48.07 | 56.3% |
| Denmark | 3005 | 50.17 | 55.5% |
| Dominican Republic | 3000 | 41.73 | 57.3% |
| Ecuador | 3000 | 40.10 | 60.6% |
| Egypt | 3000 | 39.60 | 47.0% |
| El Salvador | 3000 | 38.54 | 56.1% |
| Estonia | 3000 | 52.19 | 58.4% |
| Ethiopia | 3000 | 32.44 | 59.0% |
| Finland | 3000 | 57.46 | 53.8% |
| France | 3000 | 48.30 | 53.1% |
| Gabon | 3000 | 33.87 | 48.6% |
| Gambia | 1000 | 34.89 | 61.2% |
| Georgia | 3000 | 49.02 | 55.8% |
| Germany | 4000 | 48.21 | 51.8% |
| Ghana | 3000 | 33.12 | 46.4% |
| Greece | 3000 | 50.57 | 54.8% |
| Guatemala | 3000 | 36.11 | 59.3% |
| Guinea | 3000 | 35.91 | 48.1% |
| Haiti | 1512 | 37.53 | 52.4% |
| Honduras | 3000 | 37.85 | 58.6% |
| Hong Kong | 2012 | 44.95 | 52.8% |
| Hungary | 3000 | 53.64 | 57.9% |
| Iceland | 1625 | 48.50 | 56.3% |
| India | 9000 | 37.03 | 48.3% |
| Indonesia | 3000 | 38.17 | 59.8% |
| Iran | 3001 | 38.56 | 51.3% |
| Iraq | 3020 | 34.88 | 36.6% |
| Ireland | 3000 | 46.06 | 51.8% |
| Israel | 3000 | 42.44 | 51.1% |
| Italy | 3000 | 48.56 | 54.3% |
| Ivory Coast | 3000 | 33.88 | 37.6% |
| Jamaica | 504 | 43.74 | 56.5% |
| Japan | 3008 | 57.91 | 52.4% |
| Jordan | 3012 | 37.02 | 56.0% |
| Kazakhstan | 3000 | 41.34 | 57.6% |
| Kenya | 3000 | 31.95 | 52.1% |
| Kosovo | 3000 | 40.25 | 53.6% |
| Kuwait | 4000 | 36.65 | 28.3% |
| Kyrgyzstan | 3000 | 41.67 | 64.5% |
| Laos | 1000 | 38.22 | 58.1% |
| Latvia | 3022 | 49.58 | 64.0% |
| Lebanon | 3000 | 39.65 | 50.8% |
| Lesotho | 2000 | 41.77 | 58.9% |
| Liberia | 3000 | 32.91 | 58.2% |
| Libya | 3014 | 34.34 | 33.3% |
| Lithuania | 3000 | 46.60 | 57.2% |
| Luxembourg | 3000 | 46.03 | 50.2% |
| Macedonia | 3056 | 47.52 | 53.8% |
| Madagascar | 3000 | 36.95 | 56.6% |
| Malawi | 3000 | 33.15 | 61.8% |
| Malaysia | 1002 | 36.81 | 52.0% |
| Mali | 3000 | 34.83 | 49.5% |
| Malta | 3017 | 48.73 | 52.8% |
| Mauritania | 3000 | 34.54 | 42.4% |
| Mauritius | 2000 | 45.36 | 53.9% |
| Mexico | 3031 | 40.94 | 55.6% |
| Moldova | 3000 | 42.67 | 53.9% |
| Mongolia | 3000 | 40.25 | 56.0% |
| Montenegro | 3000 | 45.16 | 51.5% |
| Morocco | 4064 | 40.05 | 59.5% |
| Mozambique | 2000 | 33.50 | 51.6% |
| Myanmar | 3640 | 43.19 | 60.5% |
| Namibia | 1000 | 30.86 | 67.3% |
| Nepal | 3000 | 37.43 | 62.3% |
| Netherlands | 3004 | 50.78 | 47.6% |
| New Zealand | 3012 | 55.75 | 57.9% |
| Nicaragua | 3000 | 38.47 | 57.5% |
| Niger | 3000 | 33.12 | 44.5% |
| Nigeria | 3000 | 31.71 | 44.0% |
| Northern Cyprus | 2000 | 39.83 | 42.8% |
| Norway | 4005 | 52.77 | 49.4% |
| Pakistan | 3600 | 34.54 | 50.0% |
| Palestine | 3000 | 37.48 | 55.7% |
| Panama | 3000 | 43.97 | 58.9% |
| Paraguay | 2000 | 43.14 | 51.5% |
| Peru | 3000 | 40.15 | 58.2% |
| Philippines | 3000 | 40.79 | 56.0% |
| Poland | 3000 | 47.76 | 58.7% |
| Portugal | 3029 | 48.00 | 52.7% |
| Qatar | 1000 | 34.67 | 32.5% |
| Romania | 3003 | 54.32 | 58.2% |
| Russia | 6000 | 45.75 | 67.5% |
| Rwanda | 3000 | 35.01 | 55.3% |
| Saudi Arabia | 3014 | 31.09 | 39.9% |
| Senegal | 3000 | 33.47 | 49.5% |
| Serbia | 3000 | 48.64 | 54.4% |
| Sierra Leone | 3000 | 34.61 | 55.9% |
| Singapore | 3000 | 46.69 | 55.1% |
| Slovakia | 3000 | 48.60 | 58.2% |
| Slovenia | 3002 | 52.02 | 54.4% |
| Somalia | 2191 | 32.86 | 61.6% |
| South Africa | 3000 | 34.26 | 50.1% |
| South Korea | 3000 | 50.04 | 46.6% |
| South Sudan | 3000 | 31.94 | 52.7% |
| Spain | 3000 | 43.60 | 51.5% |
| Sri Lanka | 2166 | 44.71 | 59.1% |
| Sweden | 3000 | 53.42 | 51.6% |
| Switzerland | 2501 | 50.41 | 52.3% |
| Syria | 1002 | 33.07 | 52.0% |
| Taiwan | 3000 | 45.05 | 53.9% |
| Tajikistan | 3000 | 36.14 | 57.1% |
| Tanzania | 3000 | 35.19 | 57.4% |
| Thailand | 3000 | 46.46 | 61.3% |
| Togo | 3000 | 32.64 | 44.0% |
| Trinidad and Tobago | 504 | 47.73 | 55.6% |
| Tunisia | 3002 | 38.94 | 51.1% |
| Turkey | 3003 | 39.27 | 50.3% |
| Turkmenistan | 3000 | 35.97 | 48.2% |
| Uganda | 3000 | 31.89 | 55.2% |
| Ukraine | 3000 | 48.45 | 67.0% |
| United Arab Emirates | 6608 | 35.58 | 38.0% |
| United Kingdom | 3000 | 46.71 | 52.7% |
| United States | 3064 | 52.73 | 48.3% |
| Uruguay | 3000 | 49.40 | 62.3% |
| Uzbekistan | 3000 | 39.89 | 63.1% |
| Venezuela | 3000 | 42.78 | 59.1% |
| Vietnam | 3041 | 42.93 | 55.4% |
| Yemen | 3000 | 34.57 | 50.0% |
| Zambia | 3000 | 32.22 | 58.7% |
| Zimbabwe | 3000 | 36.84 | 62.9% |
| Total | 457129 | 41.72 | 53.4% |
