# Supplementary material for: A multidimensional understanding of prosperity and well-being at country level: Data-driven explorations
Source: PLoS One. 2019 Oct 9;14(10):e0223221. doi: 10.1371/journal.pone.0223221 (PMC6785080; doi:10.1371/journal.pone.0223221)
Supplement: S2 Table — (DOCX) [file pone.0223221.s005.docx]

| **Table S2**  ***Gallup Items Used in the Study*** | | |
| --- | --- | --- |
| Variable | Item | Response format |
| Life satisfaction (ladder) | Please imagine a ladder with steps numbered from zero at the bottom to ten at the top. The top of the ladder represents the best possible life for you and the bottom of the ladder represents the worst possible life for you. On which step of the ladder would you say you personally feel you stand at this time? | 00 Worst Possible  10 Best possible |
| Future life satisfaction | On which step of the ladder would you say you personally feel you stand at this time? | 00 Worst Possible  10 Best possible |
| Happiness | Did you experience the following feelings during a lot of the day yesterday? How about happiness? | 1 Yes 2 No |
| Satisfaction these days | All things considered, how satisfied are you with your life as a whole these days? | 00 Dissatisfied  10 Satisfied |
| Purpose in life | Do you feel your life has an important purpose or meaning? | 1 Yes 2 No |
| Enjoyment | Did you experience the following feelings during a lot of the day yesterday? How about Enjoyment? | 1 Yes 2 No |
| Worry | Did you experience the following feelings during a lot of the day yesterday? How about Worry? | 1 Yes 2 No |
| Sadness | Did you experience the following feelings during a lot of the day yesterday? How about Sadness? | 1 Yes 2 No |
| Stress | Did you experience the following feelings during a lot of the day yesterday? How about Stress? | 1 Yes 2 No |
| Anger | Did you experience the following feelings during a lot of the day yesterday? How about Anger? | 1 Yes 2 No |
| Smile/laughter | Did you smile or laugh a lot yesterday? | 1 Yes 2 No |
| Friendship opportunities | In the city or area where you live, are you satisfied or dissatisfied with the opportunities to meet people and make friends? | 1 Satisfied  2 Dissatisfied |
| Well-rested | Did you feel well-rested yesterday? | 1 Yes 2 No |
| *Note*. All items had also two other response options: “Don’t know” and “Refuse to answer”*.* | | |
