# Supplementary material for: A multidimensional understanding of prosperity and well-being at country level: Data-driven explorations
Source: PLoS One. 2019 Oct 9;14(10):e0223221. doi: 10.1371/journal.pone.0223221 (PMC6785080; doi:10.1371/journal.pone.0223221)
Supplement: S4 Table — (DOCX) [file pone.0223221.s007.docx]

| **Table S4**  ***Global Regions (Gallup’s Categorization)*** | |
| --- | --- |
| Region | Country |
| European Union | Austria, Belgium, Bulgaria, Croatia, Cyprus, Czech Republic, Denmark, Estonia, Finland, France, Germany, Greece, Hungary, Ireland, Italy, Latvia, Lithuania, Luxembourg, Malta, Netherlands, Poland, Poland, Romania, Slovakia, Slovenia, Spain, Sweden, United Kingdom |
| Europe-Other | Albania, Bosnia Herzegovina, Iceland, Kosovo, Macedonia, Montenegro, Northern Cyprus, Norway, Serbia, Switzerland |
| Commonwealth of Independent States | Armenia, Azerbaijan, Belarus, Georgia, Kazakhstan, Kyrgyzstan, Moldova, Nagorno Karabakh, Russia, Tajikistan, Turkmenistan, Ukraine, Uzbekistan |
| Southeast Asia | Cambodia, Indonesia, Laos, Malaysia, Myanmar, Philippines, Singapore, Thailand, Vietnam |
| South Asia | Afghanistan, Bangladesh, Bhutan, India, Nepal, Pakistan, Sri Lanka |
| East Asia | China, Hong Kong, Japan, Mongolia, South Korea, Taiwan |
| Latin America and the Caribbean | Argentina, Belize, Bolivia, Brazil, Chile, Colombia, Costa Rica, Cuba, Dominican Republic, Ecuador, El Salvador, Guatemala, Guyana, Haiti, Honduras, Jamaica, Mexico, Nicaragua, Panama, Paraguay, Peru, Puerto Rico, Suriname, Trinidad and Tobago, Uruguay, Venezuela |
| Northern America | Canada, United States |
| Australia, New Zealand | Australia, New Zealand |
| Middle East and North Africa | Algeria, Bahrain, Egypt, Iran, Iraq, Israel, Jordan, Kuwait, Lebanon, Libya, Morocco, Oman, Palestine, Qatar, Saudi Arabia, Syria, Tunisia, Turkey, United Arab Emirates, Yemen |
| Sub-Saharan Africa | Angola, Benin, Botswana, Burkina Faso, Burundi, Cameroon, Central African Republic, Chad, Comoros, Congo Brazzaville, Congo Kinshasa, Djibouti, Ethiopia, Gabon, Ghana, Guinea, Ivory Coast, Kenya, Lesotho, Liberia, Madagascar, Malawi, Mali, Mauritania, Mauritius, Mozambique, Namibia, Niger, Nigeria, Rwanda, Senegal, Sierra Leone, Somalia, Somaliland, South Africa, South Sudan, Sudan, Swaziland, Tanzania, Togo, Uganda, Zambia, Zimbabwe |
